# Therapeutic Adenovirus Vaccine Combined Immunization with IL-12 Induces Potent CD8^+^ T Cell Anti-Tumor Immunity in Hepatocellular Carcinoma

**DOI:** 10.3390/cancers14184512

**Published:** 2022-09-17

**Authors:** Yanyan Zheng, Zheng Lu, Jiage Ding, Nan Jiang, Jiawei Wang, Jie Yang, Jingyuan Song, Hongliang Chen, Lin Fang, Huizhong Li, Hui Tian, Gang Wang, Junnian Zheng, Dafei Chai

**Affiliations:** 1 Cancer Institute, Xuzhou Medical University, Xuzhou 221002, China; 2 Center of Clinical Oncology, Affiliated Hospital of Xuzhou Medical University, Xuzhou 221002, China; 3 Jiangsu Center for the Collaboration and Innovation of Cancer Biotherapy, Cancer Institute, Xuzhou Medical University, Xuzhou 221002, China; 4 Department of Urology, Affiliated Hospital of Xuzhou Medical University, Xuzhou 221002, China; 5 Xuzhou Medical University School of Nursing, Xuzhou Medical University, Xuzhou 221002, China; 6 Department of Orthopedics, Affiliated Hospital of Xuzhou Medical University, Xuzhou 221002, China

**Keywords:** HCC, Ad vaccine, GPC3, IL-12, CD8^+^ DCs, multifunctional CD8^+^ T cells

## Abstract

**Simple Summary:**

Hepatocellular carcinoma is a kind of tumor with a high malignant degree and mortality rate, and there is no effective treatment method. Currently, immunotherapy has shown good prospects in treating hepatocellular carcinoma. As an important approach of immunotherapy, the vaccine has become an attractive method for tumor treatment. This study developed an adenovirus vaccine containing tumor antigen glypican-3 and adjuvant interleukin 12. The subcutaneous tumor model was intramuscularly immunized three times with vaccines at a ten-day interval. Compared with the control group, the proliferation of CD 8^+^ T cell, the induction of multifunctional CD 8^+^ T cell and dendritic cells, and cytotoxic T lymphocyte activity were significantly increased in the combined immunization group, and the growth of tumor was inhibited obviously. The therapeutic effect of the vaccine of glypican-3 and interleukin 12 mainly depends on the anti-tumor effect of CD 8^+^ T cells mediated by dendritic cells. Likewise, this vaccine also showed a good therapeutic effect in the lung metastasis model of hepatocellular carcinoma. Therefore, the adenovirus vaccine of glypican-3 and interleukin 12 might become a potential way to treat hepatocellular carcinoma.

**Abstract:**

Hepatocellular carcinoma (HCC) is one of the cancers with the highest morbidity and mortality in the world. However, clinical progress in the treatment of HCC has not shown a satisfactory therapeutic effect. Here, we have developed a novel strategy to treat HCC with an adenovirus (Ad)-based vaccine, which contains a specific antigen glypican-3 (GPC3) and an immunostimulatory cytokine IL-12. In the subcutaneous tumor model, Ad-IL-12/GPC3 vaccine was injected into muscles three times to evaluate its therapeutic effect. Compared with the control immunization group, the Ad-IL-12/GPC3 immunization group showed a significant tumor growth inhibition effect, which was confirmed by the reduced tumor volume and the increased tumor inhibition. Ad-IL-12/GPC3 co-immunization promoted the induction and maturation of CD11c^+^ or CD8^+^CD11c^+^ DCs and increased the number of tumor-infiltrating CD8^+^ T cells. Furthermore, in the Ad-IL-12/GPC3 group, the proliferation of CD8^+^ T cells, the induction of multifunctional CD8^+^ T cells, and CTL activity were significantly increased. Interestingly, the deletion of CD8^+^ T cells abolished tumor growth inhibition by Ad-IL-12/GPC3 treatment, suggesting that CD8^+^ T cell immune responses were required to eliminate the tumor. Likewise, Ad-IL-12/GPC3 vaccine also effectively inhibited lung tumor growth or metastasis by enhancing CD8^+^ DCs-mediated multifunctional CD8^+^ T cell immune responses in the lung metastasis model. Therefore, these results indicate that IL-12 combined with Ad-GPC3 vaccine co-immunization might provide a promising therapeutic strategy for HCC patients.

## 1. Introduction

Hepatocellular carcinoma (HCC) is considered to be the most common histological form of liver cancer, and it is the fifth leading cause of tumor-related death worldwide, especially in developing countries [1,2,3]. For the treatment of HCC, surgical resection, transarterial chemoembolization, radiotherapy, and liver transplantation have been used to improve the therapeutic effect and increase the patient’s survival rates. However, the limitations of these treatments usually lead to high rates of deaths, mainly due to the recurrence and metastasis of tumors [4,5]. 

HCC has always been regarded as typical cancer, and it is closely related to the inflammation caused by long-term exposure to hepatitis viruses or toxic substances [6]. It has been found that antigen presentation dysfunction leads to poor T cell activation in HCC [7,8]. Therefore, the specific niches in tumors become anergic to cancer neoantigens due to chronic hypo-responsiveness and destroyed cytotoxic response [9]. Glypican-3 (GPC3) has an extremely high expression in HCC, but it cannot be detected in normal tissue, or a minimal amount can be detected [10]. Increased expression of GPC3 can promote the proliferation and invasion of cancer cells, so it is used as a diagnostic target for tumors [11]. Due to the presentation and immunogenicity of GPC3 on the cell surface, GPC3 has attracted people’s attention as an attractive therapeutic target for immunotherapy for HCC patients [10,12]. Notably, GPC3 has become a specific antigen to induce protective immunity against HCC [13]. However, the immunogenicity of the GPC3 vaccine alone is weak, and it cannot effectively stimulate a strong anti-tumor immune response to treat HCC.

Previous studies have proved that combining tumor-cell-based vaccination and IL-12 gene therapy can modulate the tumor microenvironment, resulting in the transition from immunosuppression to immune stimulation niche [14]. IL-12 was first identified as a cytotoxic lymphocyte maturation factor, which can stimulate the proliferation of T lymphocytes and NK cells. These cells can exhibit anti-tumor response by enhancing the induction and killing ability of CTL and NK cells [15,16,17,18]. The anti-tumor effect of IL-12 involves innate immunity and specific immunity, which is realized by regulating multiple immune cells containing T lymphocytes, NK, or NKT in different tumor models. Moreover, IL-12 confers the anti-tumor effect via enhancing IFN-γ production. IFN-γ can strongly enhance the ability of phagocytes and DCs to produce IL-12, and acts as a positive feedback mechanism leading to a powerful response to kill tumor cells [19]. Therefore, IL-12 might be used as an immune adjuvant of the adenoviruses (Ad) vaccine to promote tumor-specific immune responses.

In this study, we proved the concept of combined co-immunization of IL-12 and GPC3 to treat HCC through the Ad delivery system. The anti-tumor effect of Ad-IL-12/GPC3 was evaluated in different tumor models. The results show that the Ad-IL-12/GPC3 vaccine displayed a more substantial anti-HCC effect by reprogramming the tumor microenvironment and enhancing the response of induced multifunctional CD8^+^ T cells. In addition, the application of the Ad-IL-12/GPC3 vaccine significantly prevented lung tumor metastasis mediated by similar mechanisms. Therefore, we present evidence that Ad-IL-12/GPC3 may become the future therapeutic drug for HCC.

## 2. Materials and Methods

### 2.1. Animals

Six-week-old C57BL/6J male mice were provided by the Experimental Animal Center of Xuzhou Medical University. All animals were kept under pathogen-free conditions. 

### 2.2. Cell Lines and Cell Culture 

Human embryonic kidney cell lines 293T and 293 were purchased from ATCC, and mouse cell line Hepa1-6 was purchased from Beytime (Shanghai, China). They were grown in a DMEM (Gibco, Waltham, MA, USA; Invitrogen, Waltham, MA, USA) medium containing 10% fetal bovine serum (FBS, ExCell Bio, Shanghai, China), 1% penicillin (Sangon Biotech, Shanghai, China) and streptomycin (Sangon Biotech), at 37 °C, 5% CO_2_ incubator. To establish the Hepa1-6 cell line expressing hGPC3, the sequence of hGPC3 was cloned by EcoRI (Takara Bio, Dalian, China) and BamHI (Takara Bio) and inserted into the pCDH-CMV-MCS-EF1α-Puro vector. To package lentivirus, pCDH-hGPC3 or pCDH vector was co-transfected with psPAX2 and pMD2.G plasmid into 293T cells. The supernatant containing lentiviruses was collected after 48 h transfection and infected Hepa1-6 cells. Twenty-four hours after infection, cells were screened with puromycin, and a stable hGPC3-Hep1-6 cell line was established.

### 2.3. Construction of Plasmid

The Ad vector was constructed as follows. The coding sequence of human GPC3 was amplified by PCR from the pcDNA3.1-hGPC3 (Miaoling Bio, Wuhan, China) using primers of GPC3 (Forward primer, 5′-TTGAATTCGCCACCATGGCCGGGACCGTGCGCACCGCGTGC-3′; Reverse primer, 5′-TGCGGATCCTCAGTGCACCAGGAAGAAGAAGCACAC-3′). After purification and obtaining the target product, it was subcloned into the pCA13 vector using EcoRI and BamHI sites. In addition, the vector pCA13-IL-12 was generously donated by Professor Lin Fang. Then, the shuttle vector expressing the antigen GPC3 or IL-12 was co-transfected with pPE3 plasmid into 293 cells. The cytopathic effect of cultures was monitored continuously. The recombinant Ads were separated and purified by ultracentrifugation with cesium chloride. 293 cells were used to measure the virus titer by plaque assay.

### 2.4. PCR Assay

A genome extraction kit (QIAGEN) was used to isolate DNA from target cells by the freeze-thaw cycle. The primers are described as follows: The primers for GPC3 were mentioned above, and the primers for IL12 were below (Forward primer, 5′-TTAAGCTTATGGCTCCCCTGTGCCCCAGC-3′; Reverse primer, 5′-TTGATGGCCTGGAACTCTGTC-3′). PCR products were amplified from Ad-GPC3, Ad-IL-12, or control infected cells, and electrophoresis was carried out on 1.5% agarose gel. Then, the gel image was captured under a gel imaging system (FireReader).

### 2.5. Western Blotting

Protein was extracted by treating with RIPA buffer containing protease inhibitor. After quantification, an equal amount of protein from the Ad-GPC3 group, Ad-IL-12 group, or control group was added to the SDS sample buffer, and then protein was denatured by heating. The protein was separated by SDS polyacrylamide gel electrophoresis and transferred into PVDF membranes. Then the membranes were blocked with skim milk. After blocking, the membranes were separately incubated with the primary antibodies (GPC3, Proteintech; IL-12, Sino Biological, Beijing, China) overnight at 4 °C. After incubation, the membranes were washed with TBST. Then, the target proteins were revealed by the ECL WB detection system (Beyotime, Shanghai, China). 

### 2.6. Animal Models and Vaccine Immunization 

To study the therapeutic effect of this vaccine, mice were injected subcutaneously with 5 × 10^6^ over-expressing hGPC3-Hepa1-6 cells. On the 7th day after the tumor challenge, mice were randomly divided into four groups, and 2 × 10^8^ units of Ad-control (Ad-Ctrl), Ad-IL-12, Ad-GPC3, or Ad-IL-12/GPC3 vaccine was injected intramuscularly respectively. Meanwhile, the animals injected with the same volume of PBS were used as negative controls. To ensure the same amount of adenovirus, animals immunized with Ad-Ctrl, Ad-IL12, or Ad-GPC3 were injected with another control vaccine. All animals were vaccinated three times every 10 days to stimulate immune responses. After inoculation, the growth of the tumor was monitored by volume. The tumor volume was calculated as (length × width^2^)/2. After animals were sacrificed, the tumor tissues were taken out, and the tumor weight was recorded. The tumor inhibition rate was calculated by dividing the average tumor volume of Ad vaccine groups by that of the negative control group.

To establish the lung metastatic model, 200 μL-PBS dissolved Hepa1-6 cells (1 × 10^6^) were injected into C57BL/6J mice via the tail vein. The mice were randomly divided into four groups: Ad-control (Ad-Ctrl), Ad-IL-12, Ad-GPC3, or Ad-IL-12/GPC3. After inoculating tumor cells, muscle immunization with 2 × 10^8^ units of the vaccine was carried out three times on the 1st, 11th, and 21st days, respectively. At the end of the experiment, the lungs of mice were taken out, and the numbers of metastatic nodules in the lungs of mice bearing tumors were quantified. 

### 2.7. Preparation of Single-Cell Suspension

The single cell suspension was obtained by grinding the spleens in phosphate buffer through a 70 μm filter. Red blood cells were lysed through ACK buffer solution for 2 min on ice. The remaining cells were analyzed by flow cytometry or used for further culture. For the single cell suspension of tumors, at the end of the experiment, tumors were isolated immediately from mice, cut into about 1 mm^3^ small pieces, and incubated in digestion buffer (100 U/mL collagenase type I (Sigma-Aldrich, Burlington, MA, USA), 500 U/mL collagenase type IV (Sigma-Aldrich), and 0.01% DNase (Sigma-Aldrich) in RPMI-1640 medium (Gibco) at 37 °C for 30 min. At 10-min intervals, the digested cells were pipetted up and down repeatedly. After digestion, the cells were washed with medium and filtered using 70 µm mesh cell strainers to remove debris. The red blood cells in the suspensions were lysed with ACK lysis buffer. Tumor-infiltrating leukocytes (TILs) were separated from the single-cell suspensions using a 33% Percoll (VicMed, Xuzhou, China) gradient method. 

### 2.8. Flow Cytometry Analysis

Immune cell surface staining was used with the following antibodies, including anti-CD3ε PE, anti-CD4 PerCP-Cy5.5, anti-CD8α PerCP-Cy5.5, anti-NK1.1 FITC, anti-CD11b FITC, anti-CD11c APC, anti-Gr-1 PerCP-Cy5.5, anti-CD103 PE, anti-80 PE, anti-86 PE, anti-MHC-II PE, and anti-F4/80 PerCP. All of these antibodies were purchased from BioLegend (San Diego, CA, USA). For 293 cells and tumor cells, an anti-GPC3 antibody (APC-conjugated, Sino Biological) was used. The target cells were incubated with antibodies at 4 °C for 30 min. For intracellular cytokine staining, splenocytes were stimulated with 10 μg/mL hGPC3 protein for 72 h. Ionomycin (500 ng/mL, Sigma-Aldrich), PMA (50 ng/mL, Sigma-Aldrich), and Brefeldin A (5 ng/mL BFA, eBioscience, San Diego, CA, USA) were used to stimulate splenocytes at 37 °C and 5% CO_2_ for the last 5 h. The cells were firstly stained with antibodies against CD8α (PerCP-Cy5.5) and then conducted using intracellular staining (IFN-γ, APC; TNF-α, FITC; IL-2, PE). For IL-12 staining, 293 cells were performed using intracellular staining with IL-12/IL-23p40 (PE, BioLegend). Flow cytometry analysis was performed using BD FACS CantoII (BD Biosciences, Shanghai, China) or Cytek^®^ Northern Lights (Cytek Biosciences, Fremont, CA, USA) and analyzed by FlowJo software (Tree Star Inc., San Francisco, CA, USA).

### 2.9. Determination of CD8^+^ T Lymphocyte Proliferation 

The spleen cells from immunized animals were counted and spread on 48-well flat-bottomed tissue culture plate. The cell density was 4 × 10^5^ cells per well. To stimulate CD8^+^ T lymphocytes, IL-2 (50 U/mL) and hGPC3 protein (10 μg/mL) were used in cell culture. These cells were kept at 37 °C under a 5% CO_2_ incubator for five days, and the medium was changed every three days. BeyoClick™ EdU Cell Proliferation Kit with Alexa Fluor 647 (Beytime, Shanghai, China) was used to measure the growth of CD8^+^ T lymphocytes according to the operating instruction.

### 2.10. Cytotoxic T Lymphocyte (CTL)-Mediated Tumor Cell Killing Assay

Spleen cell suspensions were prepared from the immunized animals and cultured in a 5% CO_2_ incubator at 37 °C in the presence of IL-2 (50 U/mL) and hGPC3 protein (10 μg/mL). After five days in culture, the stimulated splenocytes were harvested and co-cultured with hGPC3-Hep1-6 cells. The treated splenocytes were washed and used as effector cells. hGPC3-Hepa1-6 cells were used as target cells. The ratio of the effector cells to target cells was 50:1 in a 24-well plate with a round bottom. After three days of co-culture, the whole cells were harvested and stained with anti-CD8α and anti-GPC3 antibodies. The killing effect was measured by FACS.

### 2.11. ELISPOT Assay

ELISPOT kit (eBioscience) was applied to detect the release of IFN-from lymphocytes in each group after being stimulated with hGPC3 protein (10 μg/mL) for 60 h at 37 °C under a humid incubator with 5% CO_2_. After washing, the biotinylated antibody was added and incubated. After washing the plate, streptavidin-horseradish peroxidase (HRP) was added and incubated. Then, after washing again, the AP-colorimetric substrate was added to the plate and incubated at room temperature. The spot-forming cells were captured by the ELISPOT reader system (AID).

### 2.12. In Vivo Depletion of CD8^+^ T Cell

CD8^+^ T cells were depleted, as described below. Two days before immunization, the animals in the therapeutic model received 0.5 mg of the purified anti-mouse CD8α mAb (clone 53–6.7) intraperitoneally and then twice a week for three weeks. The deletion of CD8^+^ T cells was examined in the splenic lymphocytes by FACS. 

### 2.13. IHC Staining

Tissue sections of mouse tumor tissues were immediately fixed in 4% PFA overnight for two days, dehydrated in alcohol, cleared in xylene, and then embedded in paraffin. Paraffin-embedded tumor slides were deparaffinized and subjected to heat-induced epitope retrieval using 0.01 M citrate buffer. The sections were blocked and incubated with antibodies against CD8 (eBioscience). The DAB Detection Kit (Zhongshan Biotech, Zhongshan, China) was applied for the following immunohistochemical staining. Images were randomly captured by Nikon SCLIPSS TE2000-S microscope (Nikon, Melville, NY, USA) using ACT-1 software, and the original magnification was 200 times. 

### 2.14. Statistical Analyses

Data analysis was preceded using GraphPad Prism (GraphPad Software, San Diego, CA, USA). The data are displayed as mean ± SD in the figures. Quantitative results were compared by a two-tailed independent Student’s *t*-test or one-way ANOVA between groups. A *p* values < 0.05 was considered significant (* *p* < 0.05, ** *p* < 0.01, *** *p* < 0.001, **** *p* < 0.0001).

## 3. Results

### 3.1. Preparation and Identification of Ad-IL-12/GPC3 Vaccine

**Figure 1:**
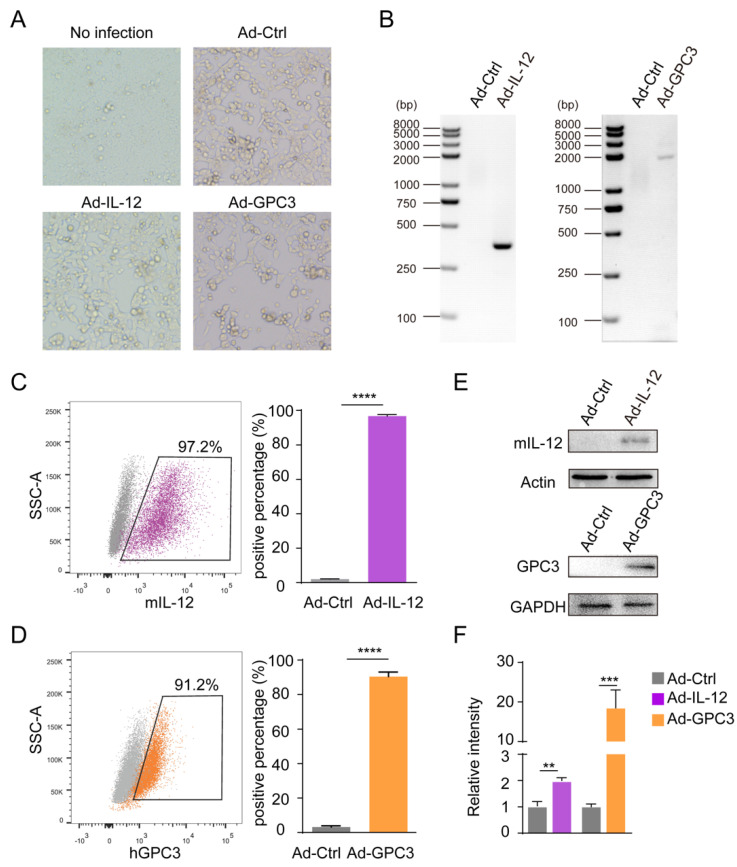
The construction and expression of Ad-IL-12 and Ad-GPC3. (**A**) The cytopathic effect (CPE) in 293 cells infected with Ad-Ctrl, Ad-IL-12, or Ad-GPC3. (**B**) Identification of the recombinant Ads by PCR. (**C**,**D**) The transduction efficiency of 293 cells infected with Ad-IL-12 or Ad-GPC3 was detected by flow cytometry, and the quantification of IL-12 or GPC3 expression was performed. (**E**) Western blot analysis of IL-12, GPC3, and GAPDH expression for muscular tissues from mice immunized with vaccines; Full pictures of the Western blots are presented in Figure S1. (**F**) Quantification of IL-12 and GPC3 expression by densitometry in (**E**); The densitometry readings/relative intensity of each band of the densitometry scans are presented in Table S1. Data are from one representative experiment of three performed and presented as the mean ± SD. The different significance was set at ** *p* < 0.01, *** *p* < 0.001, **** *p* < 0.0001.

Previous studies have shown that vaccines based on Ad vector is one of the most effective delivery platforms for gene expression in vitro and in vivo. To verify the successful construction of this system in vitro, the Ad-IL-12 and Ad-GPC3 were conducted and identified by the detection of the target protein, either IL-12 or GPC3, expressed in the infected 293 cells (Figure 1A–D). The Ad vector pCA13 was used as the corresponding control. Flow cytometry showed that after Ad-IL-12 or Ad-GPC3 infection, compared with the control cells, the expression level of IL-12 or GPC3 in 293 cells was significantly increased. In order to further confirm the in vivo expression of the vaccine, the expression of Ad-IL-12 or Ad-GPC3 was identified in the injected animal target muscles. Western blot analysis showed that the strong expression of IL-12 or GPC3 was also detected in mice treated with Ad-IL-12 or Ad-GPC3 of sufficient size compared with mice treated with Ad-Ctrl at the adequate size (Figure 1E,F). Therefore, our data indicate that Ad-IL-12/GPC3 vaccine could be effectively expressed in vivo.

### 3.2. Ad-IL12/GPC3 Vaccine Inhibits Tumor Growth and Stimulates Cytotoxic T Cells

**Figure 2:**
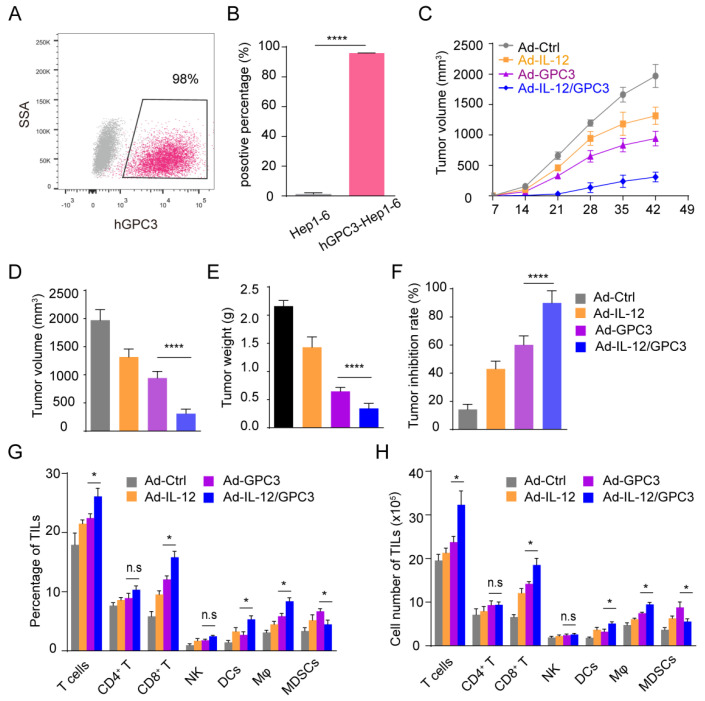
Therapeutic effects of Ad-IL-12/GPC3 vaccine in a subcutaneous tumor model. (**A**) The ratio of hGPC3 expression was determined by flow cytometry in Hep1-6 cells infected with hGPC3 lentivirus. Mice were inoculated subcutaneously at the right flank with 5 × 10^6^ hGPC3-Hep1-6 cells. Seven days later, mice were intramuscularly immunized with various vaccines. (**B**) Tumor growth was monitored once a week following the vaccination. (**C**) Tumor volumes. (**D**) Tumor weights. (**E**) Tumor inhibition rate. (**F**) Mice were sacrificed on the 42nd day after tumor inoculation. (**G**,**H**) The frequencies and total numbers of T, CD4^+^ T, CD8^+^ T, NK, DCs, macrophages, or MDSCs in TILs were analyzed. The experiments were performed with five mice per group. Data shown are representative of three experiments. Data means ± SD. The different significance was set at *, *p* < 0.05; ****, *p* < 0.001; n.s, not significant.

The cell line human GPC3 (hGPC3)-Hepa1-6 was established by hGPC3-expressing lentivirus to infect Hepa1-6 cells. Flow cytometry analysis revealed that compared with the control cells, the expression of GPC3 in hGPC3-Hep1-6 cells was significantly upregulated (Figure 2A,B). Next, hGPC3-Hep1-6 cells were used to establish subcutaneous tumor models. One week after the inoculation of hGPC3-Hepa1-6 cells, mice were immunized intramuscularly with Ad-Ctrl, Ad-IL-12, Ad-GPC3, or Ad-IL-12/GPC3 vaccine, respectively. The tumor volume and weight were monitored at the indicated time. Compared with the mice immunized with Ad-GPC3, the development of tumors in mice vaccinated with Ad-IL-12/GPC3 was inhibited. The subcutaneous tumors of operated animals were isolated and weighted to evaluate the development of tumors (Figure 2C). The tumor size and weight were dramatically reduced (Figure 2D,E). In the Ad-IL-12/GPC3 group, the tumor inhibition rate was markedly increased (Figure 2F), suggesting Ad-IL-12/GPC3 vaccine could suppress the tumor growth of the subcutaneous tumor models.

To clarify the possible mechanisms of anti-tumor effects induced by Ad-IL-12 combined immunization, we detected the proportion and quantity of several critical immune indexes, such as T cells, NK cells, DCs, macrophages, and MDSCs. Compared to other groups, Ad-IL-12/GPC3 treatment group showed an increase in DCs and macrophages, a decrease in MDSCs, and a similar number of NK cells (Figure S2). Compared with the Ad-GPC3 group, the proportion and number of cytotoxic T cells in the combined immunization group increased significantly, but there was no difference in CD4^+^ T cells (Figure 2G,H). Therefore, co-immunization with Ad-IL-12 could enhance the stimulation of immune cells in the tumor microenvironment, especially cytotoxic T cells.

### 3.3. Co-Immunization with Ad-IL-12 Promotes the Increase and Maturation of CD11c^+^ and CD8^+^CD11c^+^ DC Subsets and Induces Strong Immune Responses of CD8 T Cells 

**Figure 3:**
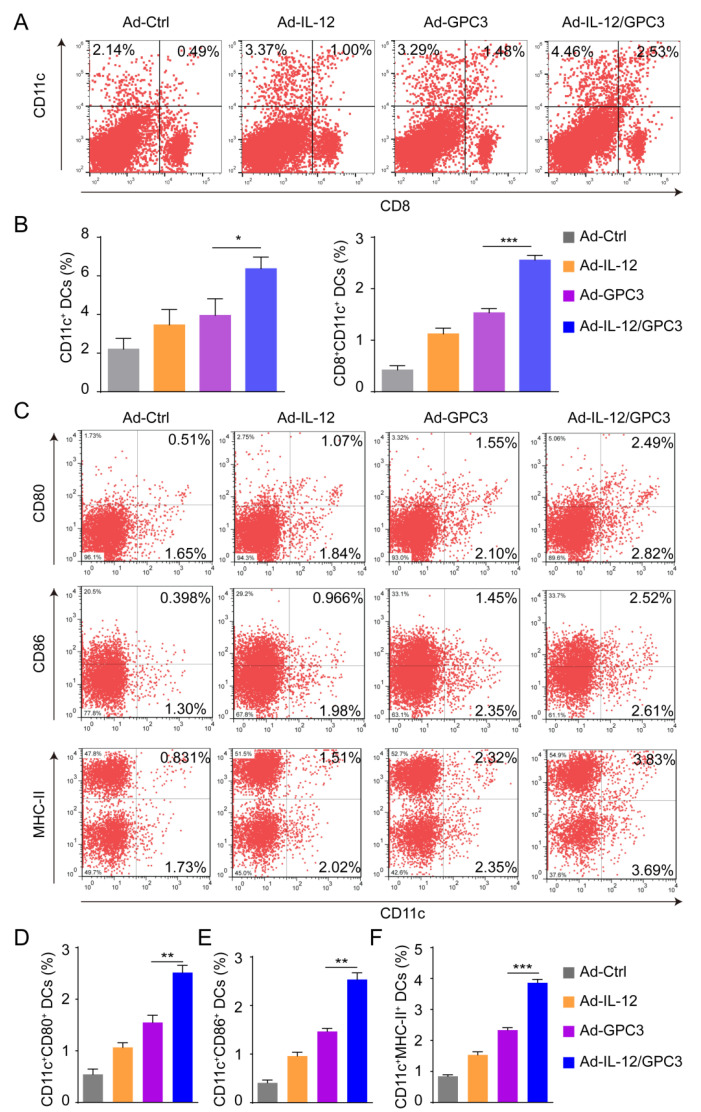
Enhancement of Ad-IL-12/GPC3 vaccine on recruitment and maturation of CD11c^+^ DCs and CD8^+^CD11c^+^ DCs subset. 42 days after tumor inoculation, spleen cells were isolated from immunized mice, and analyzed by flow cytometry. (**A**) The proportion of splenic CD11c^+^ DCs or CD8^+^CD11c^+^ DCs subset. A representative result of flow cytometry was shown. (**B**) Statistical analysis of the frequency of splenic CD11c^+^ cells. (**C**) Statistical analysis of the frequency of splenic CD8^+^CD11c^+^ subset; (**D**) the frequency of CD80, CD86, or MHC-II expression on CD11c^+^ cells in the spleen. (**E**,**F**) Statistical analysis of the frequency of CD11c^+^CD80^+^, CD11c^+^CD86^+^ or CD11c^+^MHC -II^+^ cells in spleen. Data are from one representative experiment of three performed and presented as the mean ± SD. The different significance was set at * *p* < 0.05, ** *p* < 0.01, and *** *p* < 0.001.

According to previous reports, CD8^+^CD11c^+^ DC subgroup is responsible for inducing DCs-medicated tumor-specific cytotoxic T cells to kill tumor target cells [20]. In order to further explore the possible mechanism of the anti-tumor effect of the Ad-IL-12/GPC3 vaccine, we first determined the DCs induced in the splenic cells of immunized animals. Compared with the animals immunized with the single vaccine, the proportion of CD11c^+^ DCs in the co-immunized animals was significantly increased (Figure 3A,B). Meanwhile, an increased ratio of CD8^+^CD11c^+^ DCs subset from the splenocytes was determined in the co-immunized group (Figure 3A,C). As shown in Figure 3C,D, in Ad-IL-12/GPC3 co-immunization group, the activation of markers on CD11c^+^ DCs, such as CD80, CD86, or MHC-II, was significantly up-regulated (Figure 3C,D). These results indicate that Ad-IL-12/GPC3 combined immunization promotes the increase and maturation of CD11c^+^ and CD8^+^CD11c^+^ DC subset in the HCC tumor model (Figure 3E,F).

**Figure 4:**
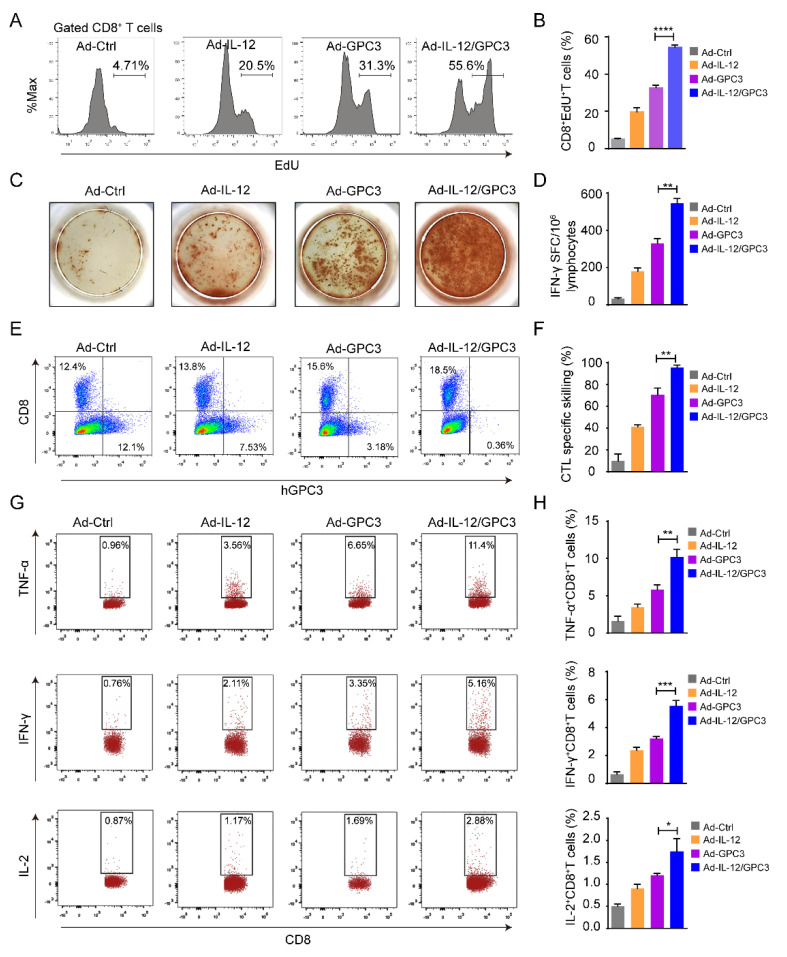
Antigen-specific CTL responses elicited by Ad-IL-12/GPC3 vaccine. (**A**) Splenocytes of immunized mice were isolated and then stimulated with hGPC3 protein. EdU incorporation assay was performed to evaluate cell proliferation ability for every group; (**B**) quantification of EdU^+^ subpopulations in CD8^+^ T cells; (**C**,**D**) antigen-specific IFN-γ-secreting T lymphocyte cells were quantified by ELISPOT assay. (**E**,**F**) CTL activity was conducted to assess the killing ability of splenocytes of immunized mice by FACS. (**G**,**H**) The splenocytes were analyzed by flow cytometry to evaluate the proportion of TNF-α^+^CD8^+^, IL-2^+^CD8^+^, and IFN-γ^+^CD8^+^ T cells. Results from one representative experiment are shown for each group of mice. Data are from one representative experiment of three performed and presented as the mean ± SD. The different significance was set at * *p* < 0.05, ** *p* < 0.01, *** *p* < 0.001 and **** *p* < 0.0001.

In most therapies, the killing ability of tumor-specific cytotoxic T cells to target cells induced by DCs is essential to evaluate the lytic functions of rejecting the formed tumor cells. To assess the immune responses after Ad-IL-12/GPC3 immunization, the proliferation potential of CD8^+^ T cells induced by GPC3 was measured by the EdU incorporation method in vitro. The co-immunized Ad-IL-12/GPC3 group showed higher cell proliferation ability than the other immunized groups (Figure 4A,B). We also assessed the function of CD8^+^ T cells expressing cytokines, including TNF-α, IL-2, and IFN-γ by ELISPOT assay or FACS. The number of GPC3-specific IFN-γ producing cells from the co-immunized animals was remarkably higher than that of other animals (Figure 4C,D). To assess the lytic function of tumor-specific cytotoxic T cells, hGPC3-Hep1-6 cells were used as target cells, and spleen lymphocytes from immunized mice were used as effector cells. Cell killing effect was detected in vitro by co-culture test. The lytic potential of lymphocytes in co-immunized animals was improved, but the cytolysis caused by other groups was significantly reduced (Figure 4E,F). The results of intracellular staining by flow cytometry revealed that compared with other animals, the proportion of CD8^+^ T cells expressing specific immune factors, such as TNF-α, IL-2, and IFN-γ in co-immunized animals, increased significantly (Figure 4G,H). These results show that Ad-IL-12/GPC3 co-immunization strongly promoted the proliferation and killing ability of functional CD8^+^ T cells induced by tumor-specific antigen GPC3. Collectively, these results indicate that co-immunization with Ad-IL-12/GPC3 promotes the increase and maturation of DCs and the immune responses of antigen-specific CD8^+^ T cells. 

### 3.4. The Multifunctional CD8^+^ T Cell Immune Responses Are Necessary for the Anti-Tumor Effect 

**Figure 5:**
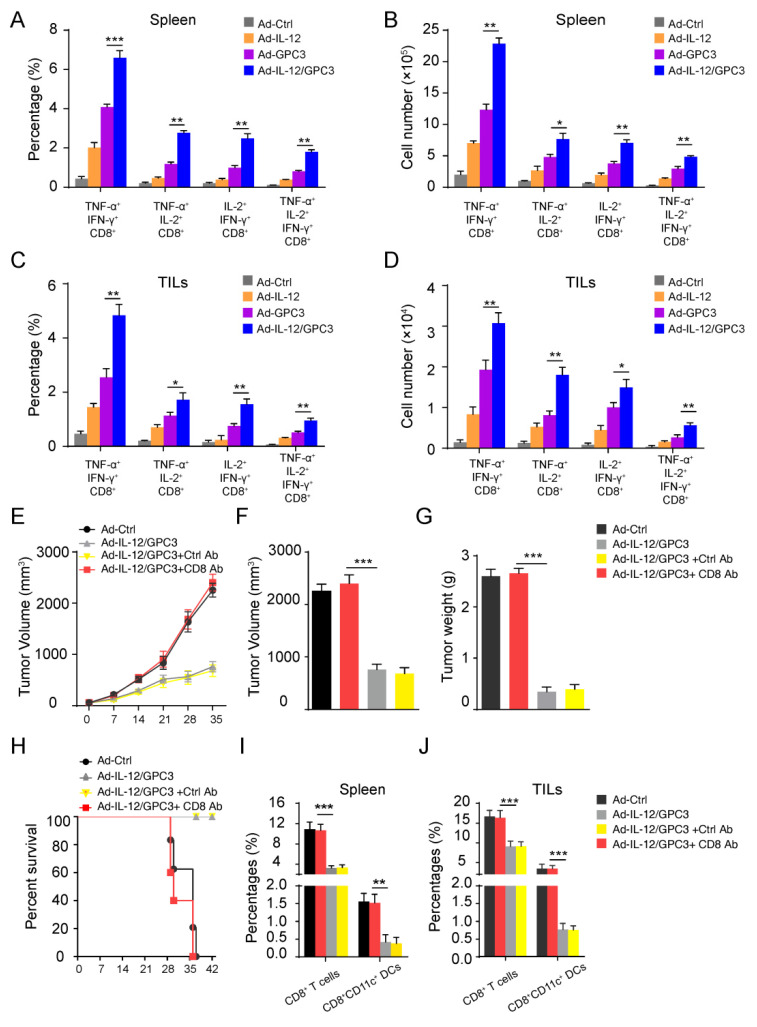
Anti-tumor response induced by Ad-IL-12/GPC3 vaccine was CD8^+^ T cell-dependent. (**A**,**B**) The percentages and amounts of cells expressing TNF-α^+^IL-2^+^, TNF-α^+^IFN-γ^+^, IL-2^+^IFN-γ^+^, and TNF-α^+^IL-2^+^IFN-γ^+^ by gating on CD8^+^ T cells from splenocytes stimulated with 10 μg/mL GPC3 protein for 72 h, with 500 ng/mL ionomycin (Sigma-Aldrich) and 50 ng/mL PMA plus 5 ng/mL BFA for the last 5 h. (**C**,**D**) CD8^+^ T cells expressing TNF-α^+^IL-2^+^, TNF-α^+^IFN-γ^+^, IL-2^+^IFN-γ^+^ or TNF-α^+^IL-2^+^IFN-γ^+^ were detected in the stimulated TILs. In the CD8 depletion group, mice were intraperitoneally injected with 0.5 mg anti-mouse CD8 mAb 2 days before the vaccine’s first administration. The antibody injection was repeated on the 5th and 12th days after the first vaccination. (**E**,**F**) Mice were sacrificed on the 42nd day after tumor inoculation in Ad-IL-12/GPC3 and Ad-IL-12/GPC3+Ctrl Ab groups, while mice in other groups were sacrificed before the 42nd day due to the heavy tumor burden and tumor volumes in four groups were measured on the 35th day after tumor inoculation. (**G**) Weights of tumor; (**H**) survival rate. (**I**,**J**) The proportions of CD8^+^ T cells or CD8^+^CD11c^+^ cells were assessed in spleen or TILs. The experiments were performed with five mice per group. Data shown are representative of three experiments. Data, mean ± SD, * *p* < 0.05, ** *p* < 0.01, and *** *p* < 0.001.

Multifunctional CD8^+^ T lymphocytes, producing TNF-α, IL-2, and IFN-γ, are associated with immunity protection. Therefore, we tested whether tumor vaccine therapy has a protective effect. We examined the stimulation of GPC3-mediated multifunctional CD8^+^ T lymphocytes in spleen cells and tumor-infiltrating lymphocytes of tumor models immunized with various vaccines. Remarkably, the rate of two-marker-expressing CD8^+^ T lymphocytes (TNF-α^+^IL-2^+^, TNF-α^+^IFN-γ^+^, and IL-2^+^IFN-γ^+^) and three-marker-expressing CD8^+^ T lymphocytes (TNF-α^+^IL-2^+^IFN-γ^+^) were increased in co-immunized animals compared to other groups (Figure 5A–D). The result indicates that the co-immunization of the Ad-GPC3 vaccine and IL-12 could effectively stimulate GPC3-specific multifunctional CD8^+^ T lymphocytes. Therefore, we predicted that multifunctional CD8^+^ T lymphocytes are necessary for the anti-tumor effect induced by Ad-IL-12/GPC3 vaccine. 

In order to test whether the therapeutic benefit of the Ad-IL-12/GPC3 vaccine regimen in the tumor model depended on CD8^+^ T lymphocytes, we used anti-CD8 monoclonal antibodies (mAbs) to carry out a deletion test. Results show that elimination of this specificity significantly inhibited the anti-tumor effects related to Ad-IL-12/GPC3 (Figure 5E–J). This finding indicates that CD8^+^ T cells are indispensable for the efficacy of Ad-IL-12/GPC3 vaccine.

### 3.5. Ad-IL-12/hGPC3 Vaccine Inhibits Tumor Lung Metastasis by Promoting Multifunctional CD8^+^ T-Cell Responses in the Lung Metastasis Model

**Figure 6:**
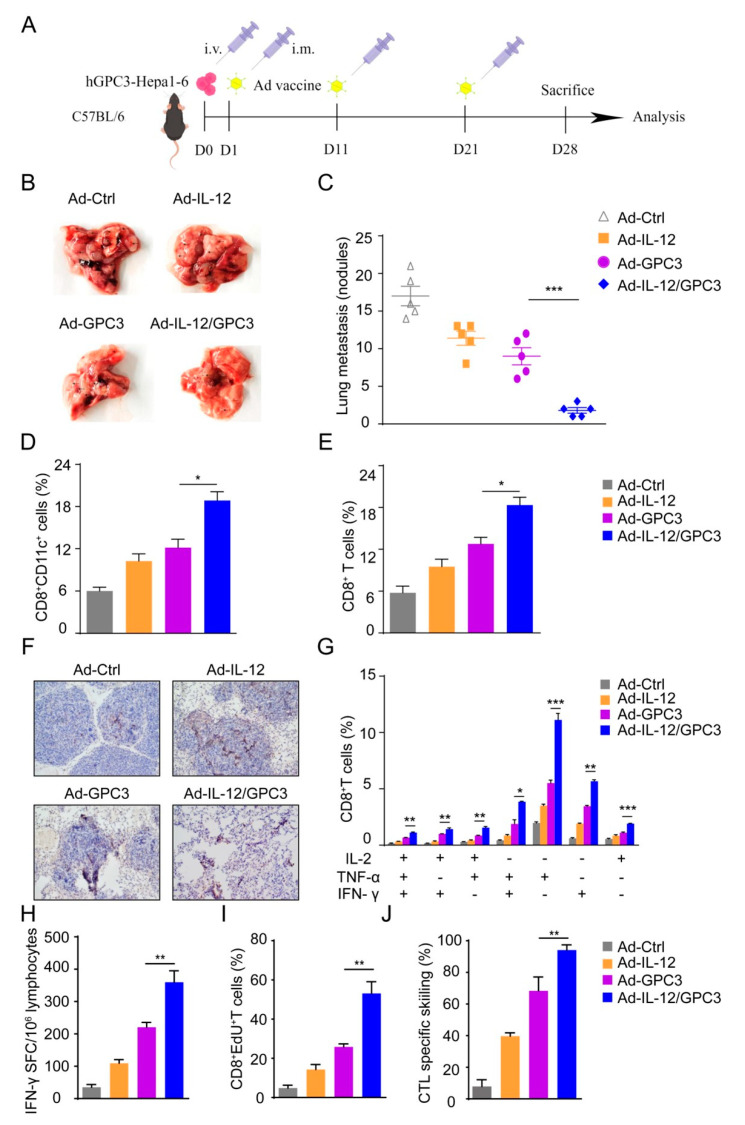
Therapeutic effects of Ad-IL-12/GPC3 vaccine in lung metastasis model. (**A**) Schematic of the timeline of the model; (**B**) the present images of the lung tumor removed from mice; (**C**) the numbers of metastatic nodules were quantified in the lung of the tumor-bearing mice; (**D**,**E**) mice were sacrificed on day 28 after tumor inoculation, and the frequencies of CD8^+^CD11c^+^ cells and CD8^+^ T cells in the lung tumor tissues of each vaccine groups were analyzed. (**F**) Immunochemistry staining for CD8^+^ T cells in lung tumor tissues. (**G**) The percentages of TNF-α^+^IL-2^+^CD8^+^ T cells, TNF-α^+^IFN-γ^+^CD8^+^ T cells, IL-2^+^IFN-γ^+^CD8^+^ T cells or TNF-α^+^IL-2^+^IFN-γ^+^CD8^+^ T cells were detected in splenocytes stimulated with GPC3 protein (10 μg/mL) for 72 h, and stimulated with 500 ng/mL ionomycin and 50 ng/mL PMA plus 5 ng/mL BFA for the last 5 h. (**H**) Antigen-specific IFN-γ-secreting T lymphocyte cells were detected by ELISPOT. (**I**) Quantification of EdU^+^CD8^+^ subsets in CD8^+^ T cells. (**J**) CTL activity. The experiments were performed with five mice per group. Data shown are representative of three experiments. Data, mean ± SD, * *p* < 0.05, ** *p* < 0.01, and *** *p* < 0.001.

In order to evaluate the therapeutic potential of Ad-GPC3 vaccine and IL-12 co-immunization in preventing tumor metastasis in the animal model, we established a model of lung metastasis of liver cancer in mice. Inoculating hGPC3-Hep1-6 cells, animals were immunized with Ad-IL-12/GPC3 or the control vaccine by intramuscular injection. Twenty-one days after inoculation with hGPC3-Hep1-6, animals were sacrificed. The lungs were isolated from mice, and then the metastasis was recorded. Compared with other immunization groups, the number of lung metastases was generally reduced in Ad-IL-12/GPC3 immunization group (Figure 6A–C). During the experiments, the weights of the animals were also recorded, and there was no significant difference between the groups. This finding indicates that this combined immunization is safe, which is an essential indicator in vaccine-based therapy. 

Cytotoxic CD8^+^ T lymphocytes can hardly be detected in tumor tissues, and it shows impaired functions in patients with HCC, accompanied by lymphocyte reduction. These changes are attributed to the immunosuppressive tumor microenvironment and have an adverse effect on tumor prognosis, which indicates that preventing T cell loss is helpful to treatment results. To ensure that activated T cells could be collected in the tumor after treatment, we measured the ratio of DCs and CD8^+^ T cells. Compared to other groups, a remarkable rise in infiltrating CD8^+^CD11c^+^ DCs or CD8^+^ T lymphocytes in tumor tissues was found in co-immunization animals (Figure S3 and Figure 6D,E). The result of immunohistochemical staining showed that the number of tumor-infiltrating CD8^+^ T cells in the Ad-IL-12/GPC3 group was significantly higher than that in other groups (Figure 6F). These results indicate that co-immunization with IL-12 promotes multifunctional CD8^+^ T cell responses and inhibits the metastasis in lung metastasis models of liver cancer.

In order to further verify the function of CD8 T cells treated with Ad-IL-12/GPC3 vaccine, intracellular staining was conducted on splenic cells after stimulation with GPC3 protein. The ratios of one marker (TNF-α, IL-2, and IFN-γ), two markers (TNF-α^+^IL-2^+^, TNF-α^+^IFN-γ^+^, and IL-2^+^IFN-γ^+^), and three markers (TNF-α^+^IL-2^+^ IFN-γ^+^) producing CD8^+^ T lymphocytes in the animals immunized with Ad-IL12/hGPC3 was dramatically increased compared to that from others. In addition, the ratio of infiltrating multifunctional CD8^+^ T cells in tumor tissues was significantly higher in the co-immunization animals compared to other groups. CD8^+^ T cells expressing two- and three-marker displayed the most significant rise in co-immunized animals compared with other vaccine-immunized mice (Figure 6G). This finding indicates that immunization with Ad-IL-12/GPC3 significantly promotes the stimulation of GPC3-specific multifunctional CD8^+^ T cells.

Meanwhile, compared with other animals, the increase of IFN-γ-producing T cells was observed in the co-immunized animals (Figure 6H). To better understand the stimulation of CD8^+^ T lymphocytes, the proliferation of CD8^+^ T cells in spleen cells mediated by GPC3 protein was detected by the EdU method. The growth capacity of CD8^+^ lymphocytes was improved in co-immunization animals related to others (Figure 6I). The killing ability of CD8^+^ lymphocytes in the Ad-IL12/GPC3 group against tumor target cells was significantly higher than that of other groups (Figure 6J). Consequently, this observation indicates that this combined vaccine could enhance the specific anti-tumor immune response mediated by CD8^+^ T lymphocytes in the lung metastasis mice model of liver cancer. 

## 4. Discussion

Immunotherapy is growing among the interventions designed to treat tumors and is considered one of the most effective approaches [21,22,23]. A critical factor in developing a CTL-based therapy is to develop epitope-based vaccines that can trigger this response. According to research, the genetic engineering of vectors expressing virus antigens has progressed, indicating that virus-specific CTL has been an indispensable part of medicating immune response [24]. In order to optimize the therapeutic effect of the tumor vaccine, the combinations of tumor antigen and immunologic adjuvant should be explored to ensure good clinical results [25].

Adenovirus has become a competitive system for developing gene therapy and vaccine applications, which depends on the possibility of manipulating foreign fragments and stably integrating them into the Ad genome. Because of their excellent safety and effectiveness, Ad-based vectors are gradually being used in clinical and preclinical trials [26,27]. Thus, they are being explored as attractive carriers of tumor immunotherapy [24,28,29]. Here, we chose the recombinant Ad type 5 for non-replication and robust expression level of the target gene. For this study, we employed Ad-IL-12 and Ad-GPC3 to determine their anti-tumor effects on hepatoma cells. Fortunately, Ad-IL-12/Ad-GPC3 revealed ideal expression levels both in vitro and in vivo. 

According to previous research, GPC3 is now becoming an attractive TAA for developing HCC vaccines, specifically in HCC patients. However, the antibody-mediated immunological therapy by GPC3 antibodies was ineffective in treating HCC, and the reason was the specificity against cancer [30,31,32]. Therefore, the stimulation of anti-tumor CTL has become an attractive tumor therapy with a synergistic effect [33]. As our research shows, the tumor volume and weight of the animals immunized with Ad-GPC3 were reduced. In order to improve the anti-tumor effect of the immune system and the surrounding environment of tumors, vaccine-induced immunotherapy intervention has shown an optimistic approach in clinical practice [34,35]. 

To improve therapeutic efficiency, researchers have designed different strategies by upregulating the affinity of antibodies to antigens, developing a bispecific for GPC3 and the T-cell-specific antigen CD3, or using multikinase inhibitors for combination therapy [36,37,38]. In this study, to stimulate a strong immune response and functional anti-tumor effects, adenovirus vaccine vector expressing GPC3 were generated for a prime-boost regimen. Meanwhile, IL-12 was used as an adjuvant to enhance immune function. IL-12 has been proved to be a mediator in promoting Th1/Tc1 response and T cell recruitment to tumors [17,39,40,41]. In our research, Ad-GPC3 induced a neutral immune response but showed unsatisfactory results. Remarkably, IL-12 could be used as an adjuvant, significantly promoting the tumor antigen-specific immune responses to GPC3. In this study, co-immunization with IL-12 effectively increased the proportion of CD8^+^CD11c^+^ DCs subgroup and up-regulated the activation markers of DCs. In addition, compared with single Ad-IL-12 or Ad-GPC3 vaccine alone, the combined vaccine could stimulate the growth of T cells, CTL activity, and the induction of functional CD8^+^ T cell. After combined immunization, compared with Ad-IL-12 or Ad-GPC3 alone, the numbers of immune effector CD 8^+^ T cells and dendritic cells were increased in the tumor, indicating that there was a synergistic effect in stimulating immunity. Although the effector immune cells by injection of IL-12 were not the same as in other papers, it may result from different tumor cells and delivery tools [42]. In addition, it was reported that IL-12 could enhance the memory responses of CD8^+^ T cell effector and the challenge of tumor cells would be confirmed in future work [43,44]. However, these results indicate that co-immunization with IL-12 significantly promoted cytotoxic T-cell-mediated immune responses. Therefore, Ad-IL-12/GPC3 combined immunization significantly suppressed tumor development, reduced tumor weight, and improved the tumor inhibition rate of the immunized tumor model. These results indicate that IL-12 combined immunization seems to be an effective and safe approach for HCC.

Strategies focused on restoring inherent anti-tumor immunity to reverse the milieu beneficial to the development of HCC. Although the anti-GPC3 antibody was used to block the signal transduction pathway to suppress tumor cells and showed good tolerance, it is almost impossible to eliminate the tumor in the mouse model (14, 15). Moreover, in the clinical trials, limited responses of HCC patients were observed (10, 16). Therefore, immunotherapy using a specific antibody against a GPC3 antibody may be ineffective for HCC treatment because of the lack of a tumor-specific CTL response (15, 17, 18). Based on these observations, anti-tumor CTL should be improved in cooperation. Due to the optimization of anti-tumor immunity and the reprogramming of the tumor microenvironment, vaccine-mediated immunotherapy strategy is expected to be applied in clinic.

Our results demonstrated that this co-immunization vaccine suppressed the tumor development of multiple hepatocarcinoma by generating anti-tumor immune response via cytotoxic T cell provoked by CD8^+^CD11c^+^ DCs, suggesting that strengthening co-immunization could stimulate CD8^+^ T cell immune responses ideally. To better understand the importance of CD8^+^ T cells under the condition of tumor burden, we used mAb against CD8^+^ T cells for deletion experiments. In the animals injected with Ad-IL-12/GPC3, the therapeutic effect was destroyed, which indicates that this co-immunization vaccine requires CD8^+^ T cell responses provoked by CD8^+^CD11c^+^ DCs. In addition, we further detected the proportion of CD4^+^ T cells in vaccine-immunized animals, but there was no significant difference among the groups. However, CD4^+^ T cells could be detected in every group of tumor tissues, and CD4^+^ T cells also play an essential role in inducing and stimulating tumor-specific CD8^+^ T cells.

## 5. Conclusions

In conclusion, our research shows that it is adequate to stimulate cytotoxicity T cell responses in mice by using HCC-specific antigen-GPC3 and IL-12 adjuvant recombinant Ad. This co-immunization in our study promoted the immunogenicity of HCC-specific antigen-GPC3 and efficiently suppressed tumor development of HCC. This finding indicates that the antineoplastic effects provoked by this co-immunized vaccine are necessary to induce cytotoxic T cell functionally relaying on CD8^+^CD11c^+^ DCs. Therefore, Ad-IL-12/GPC3 vaccines might be applied to treat HCC.

## Data Availability

Data is contained within the article.

## Supplementary Materials

The following supporting information can be downloaded at: https://www.mdpi.com/article/10.3390/cancers14184512/s1, Figure S1: The whole blot (uncropped blots) showing all the bands with all molecular weight markers. Figure S2: Tumor infiltration of immune cells was detected by flow cytometry. Figure S3: The percentages of CD8^+^CD11c^+^ cells were detected in DCs from lung tumor by flow cytometry. Table S1: The densitometry readings/relative intensity of each band of the densitometry scans.

## Abbreviation

HCC: Hepatocellular carcinoma; GPC3, Glypican-3; IL-12, Interleukin 12; Ad, Adenovirus; mAb, Monoclonal antibody; TILs, Tumor-infiltrating lymphocytes; DCs, Dendritic cells; IHC, Immunohistochemistry; HE, Hematoxylin Eosin; ELISPOT, Enzyme-linked immunosorbent spot.

## References

[1] El-Serag H.B., Rudolph K.L. (2007). Hepatocellular carcinoma: Epidemiology and molecular carcinogenesis. Gastroenterology.

[2] Altekruse S.F., McGlynn K.A., Reichman M.E. (2009). Hepatocellular carcinoma incidence, mortality, and survival trends in the United States from 1975 to 2005. J. Clin. Oncol..

[3] Singal A.G., Lampertico P., Nahon P. (2020). Epidemiology and surveillance for hepatocellular carcinoma: New trends. J. Hepatol..

[4] Clavien P.-A., Petrowsky H., DeOliveira M.L., Graf R. (2007). Strategies for safer liver surgery and partial liver transplantation. New Engl. J. Med..

[5] Raza A., Sood G.K. (2014). Hepatocellular carcinoma review: Current treatment, and evidence-based medicine. World. J. Gastroenterol..

[6] Sherman M. (2005). Hepatocellular carcinoma: Epidemiology, risk factors, and screening. Semin. Liver Dis..

[7] Tatsumi T., Takehara T., Katayama K., Mochizuki K., Yamamoto M., Kanto T., Sasaki Y., Kasahara A., Hayashi N. (1997). Expression of costimulatory molecules B7-1 (CD80) and B7-2 (CD86) on human hepatocellular carcinoma. Hepatology.

[8] Pillarisetty V.G., Shah A.B., Miller G., Bleier J.I., DeMatteo R.P. (2004). Liver dendritic cells are less immunogenic than spleen dendritic cells because of differences in subtype composition. J. Immunol..

[9] Jiang Y., Li Y., Zhu B. (2015). T-cell exhaustion in the tumor microenvironment. Cell Death Dis..

[10] Nakatsura T., Yoshitake Y., Senju S., Monji M., Komori H., Motomura Y., Hosaka S., Beppu T., Ishiko T., Kamohara H. (2003). Glypican-3, overexpressed specifically in human hepatocellular carcinoma, is a novel tumor marker. Biochem. Biophys. Res. Commun..

[11] Capurro M., Wanless I.R., Sherman M., Deboer G., Shi W., Miyoshi E., Filmus J. (2003). Glypican-3: A novel serum and histochemical marker for hepatocellular carcinoma. Gastroenterology.

[12] Guo M., Zhang H., Zheng J., Liu Y. (2020). Glypican-3: A New Target for Diagnosis and Treatment of Hepatocellular Carcinoma. J. Cancer.

[13] Tsuchiya N., Yoshikawa T., Fujinami N., Saito K., Mizuno S., Sawada Y., Endo I., Nakatsura T. (2017). Immunological efficacy of glypican-3 peptide vaccine in patients with advanced hepatocellular carcinoma. Oncoimmunology.

[14] Jarosz-Biej M., Smolarczyk R., Cichon T., Kulach N., Czapla J., Matuszczak S., Szala S. (2015). Combined Tumor Cell-Based Vaccination and Interleukin-12 Gene Therapy Polarizes the Tumor Microenvironment in Mice. Arch. Immunol. Ther. Exp..

[15] Lasek W., Zagozdzon R., Jakobisiak M. (2014). Interleukin 12: Still a promising candidate for tumor immunotherapy?. Cancer Immunol. Immunother..

[16] Stern A.S., Podlaski F.J., Hulmes J.D., Pan Y.C., Quinn P.M., Wolitzky A.G., Familletti P.C., Stremlo D.L., Truitt T., Chizzonite R. (1990). Purification to homogeneity and partial characterization of cytotoxic lymphocyte maturation factor from human B-lymphoblastoid cells. Proc. Natl. Acad. Sci. USA.

[17] Trinchieri G. (2003). Interleukin-12 and the regulation of innate resistance and adaptive immunity. Nat. Rev. Immunol..

[18] Kobayashi M., Fitz L., Ryan M., Hewick R.M., Clark S.C., Chan S., Loudon R., Sherman F., Perussia B., Trinchieri G. (1989). Identification and purification of natural killer cell stimulatory factor (NKSF), a cytokine with multiple biologic effects on human lymphocytes. J. Exp. Med..

[19] Weiss J.M., Subleski J.J., Wigginton J.M., Wiltrout R.H. (2007). Immunotherapy of cancer by IL-12-based cytokine combinations. Expert Opin. Biol. Ther..

[20] Li L., Kim S., Herndon J.M., Goedegebuure P., Belt B.A., Satpathy A.T., Fleming T.P., Hansen T.H., Murphy K.M., Gillanders W.E. (2012). Cross-dressed CD8α^+^/CD103^+^ dendritic cells prime CD8^+^ T cells following vaccination. Proc. Natl. Acad. Sci. USA.

[21] Neves H., Kwok H.F. (2015). Recent advances in the field of anti-cancer immunotherapy. BBA Clin..

[22] McCune J.S. (2018). Rapid Advances in Immunotherapy to Treat Cancer. Clin. Pharmacol. Ther..

[23] Sun W. (2017). Recent advances in cancer immunotherapy. J. Hematol. Oncol..

[24] Rosenberg S.A., Zhai Y., Yang J.C., Schwartzentruber D.J., Hwu P., Marincola F.M., Topalian S.L., Restifo N.P., Seipp C.A., Einhorn J.H. (1998). Immunizing patients with metastatic melanoma using recombinant adenoviruses encoding MART-1 or gp100 melanoma antigens. J. Natl. Cancer Inst..

[25] Bolhassani A., Safaiyan S., Rafati S. (2011). Improvement of different vaccine delivery systems for cancer therapy. Mol. Cancer.

[26] Top F.H. (1975). Control of adenovirus acute respiratory disease in U.S. Army trainees. Yale J. Biol. Med..

[27] Chaloner-Larsson G., Contreras G., Furesz J., Boucher D.W., Krepps D., Humphreys G.R., Mohanna S.M. (1986). Immunization of Canadian Armed Forces personnel with live types 4 and 7 adenovirus vaccines. Can. J. Public Health..

[28] Stewart A.K., Lassam N.J., Quirt I.C., Bailey D.J., Rotstein L.E., Krajden M., Dessureault S., Gallinger S., Cappe D., Wan Y. (1999). Adenovector-mediated gene delivery of interleukin-2 in metastatic breast cancer and melanoma: Results of a phase 1 clinical trial. Gene. Ther..

[29] Kusumoto M., Umeda S., Ikubo A., Aoki Y., Tawfik O., Oben R., Williamson S., Jewell W., Suzuki T. (2001). Phase 1 clinical trial of irradiated autologous melanoma cells adenovirally transduced with human GM-CSF gene. Cancer Immunol. Immunother..

[30] Feng M., Gao W., Wang R., Chen W., Man Y.-G., Figg W.D., Wang X.W., Dimitrov D.S., Ho M. (2013). Therapeutically targeting glypican-3 via a conformation-specific single-domain antibody in hepatocellular carcinoma. Proc. Natl. Acad. Sci. USA..

[31] Abou-Alfa G.K., Puig O., Daniele B., Kudo M., Merle P., Park J.-W., Ross P., Peron J.-M., Ebert O., Chan S. (2016). Randomized phase II placebo controlled study of codrituzumab in previously treated patients with advanced hepatocellular carcinoma. J. Hepatol..

[32] Schmidt N., Neumann-Haefelin C., Thimme R. (2012). Cellular immune responses to hepatocellular carcinoma: Lessons for immunotherapy. Dig. Dis..

[33] Palucka K., Banchereau J. (2013). Dendritic-cell-based therapeutic cancer vaccines. Immunity.

[34] Lin C.C., Chou C.W., Shiau A.L., Tu C.F., Ko T.M., Chen Y.L., Yang B.C., Tao M.H., Lai M.D. (2004). Therapeutic HER2/Neu DNA vaccine inhibits mouse tumor naturally overexpressing endogenous neu. Mol. Ther. J. Am. Soc. Gene Ther..

[35] Lambricht L., Vanvarenberg K., de Beuckelaer A., van Hoecke L., Grooten J., Ucakar B., Lipnik P., Sanders N.N., Lienenklaus S., Preat V. (2016). Coadministration of a Plasmid Encoding HIV-1 Gag Enhances the Efficacy of Cancer DNA Vaccines. Mol. Ther..

[36] Wu X., Luo H., Shi B., Di S., Sun R., Su J., Liu Y., Li H., Jiang H., Li Z. (2019). Combined Antitumor Effects of Sorafenib and GPC3-CAR T Cells in Mouse Models of Hepatocellular Carcinoma. Mol. Ther..

[37] Du K., Li Y., Liu J., Chen W., Wei Z., Luo Y., Liu H., Qi Y., Wang F., Sui J. (2021). A bispecific antibody targeting GPC3 and CD47 induced enhanced antitumor efficacy against dual antigen-expressing HCC. Mol. Ther..

[38] Zhao J., Lin L., Luo Y., Cai Q., Jiang X., Liao C., Wei H. (2021). Optimization of GPC3-specific chimeric antigen receptor structure and its effect on killing hepatocellular carcinoma cells. Bioengineered.

[39] del Vecchio M., Bajetta E., Canova S., Lotze M.T., Wesa A., Parmiani G., Anichini A. (2007). Interleukin-12: Biological properties and clinical application. Clin. Cancer Res..

[40] Colombo M.P., Trinchieri G. (2002). Interleukin-12 in anti-tumor immunity and immunotherapy. Cytokine Growth Factor Rev..

[41] Hu J., Sun C., Bernatchez C., Xia X., Hwu P., Dotti G., Li S. (2018). T-cell Homing Therapy for Reducing Regulatory T Cells and Preserving Effector T-cell Function in Large Solid Tumors. Clin. Cancer Res..

[42] Sun Y., Yang J., Yang T., Li Y., Zhu R., Hou Y., Liu Y. (2021). Co-delivery of IL-12 cytokine gene and cisplatin prodrug by a polymetformin-conjugated nanosystem for lung cancer chemo-gene treatment through chemotherapy sensitization and tumor microenvironment modulation. Acta. Biomater..

[43] Chowdhury F.Z., Ramos H.J., Davis L.S., Forman J., Farrar J.D. (2011). IL-12 selectively programs effector pathways that are stably expressed in human CD8^+^ effector memory T cells in vivo. Blood.

[44] Halwani R., Boyer J.D., Yassine-Diab B., Haddad E.K., Robinson T.M., Kumar S., Parkinson R., Wu L., Sidhu M.K., Phillipson-Weiner R. (2008). Therapeutic vaccination with simian immunodeficiency virus (SIV)-DNA + IL-12 or IL-15 induces distinct CD8 memory subsets in SIV-infected macaques. J. Immunol..

