# Supplementary material for: Therapeutic Adenovirus Vaccine Combined Immunization with IL-12 Induces Potent CD8+ T Cell Anti-Tumor Immunity in Hepatocellular Carcinoma
Source: Cancers (Basel). 2022 Sep 17;14(18):4512. doi: 10.3390/cancers14184512 (PMC9497125; doi:10.3390/cancers14184512)

Figure S1: The whole blot (uncropped blots) showing all the bands with all molecular weight markers.

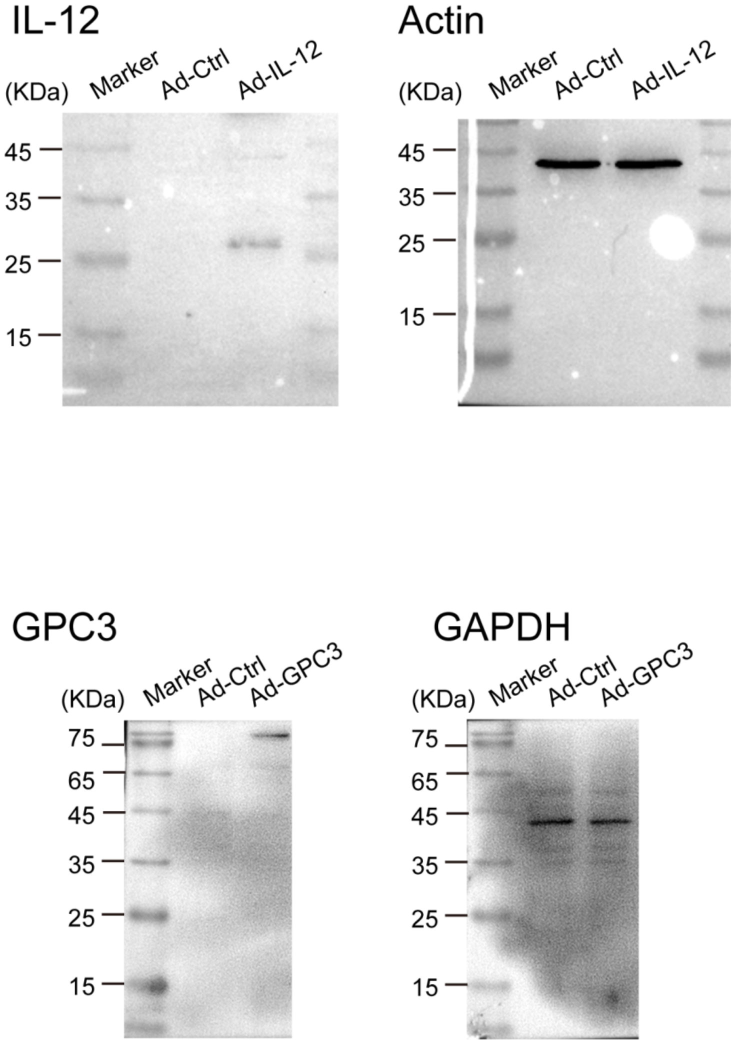

Supplement: Supplementary file 1 [file cancers-14-04512-s001.zip › Figure S1.pdf]
