# Supplementary material for: Therapeutic Adenovirus Vaccine Combined Immunization with IL-12 Induces Potent CD8+ T Cell Anti-Tumor Immunity in Hepatocellular Carcinoma
Source: Cancers (Basel). 2022 Sep 17;14(18):4512. doi: 10.3390/cancers14184512 (PMC9497125; doi:10.3390/cancers14184512)
Supplement: Supplementary file 1 [file cancers-14-04512-s001.zip › Figure S2.pdf]

Figure. S2. Tumor infiltration of immune cells was detected by flow cytometry.

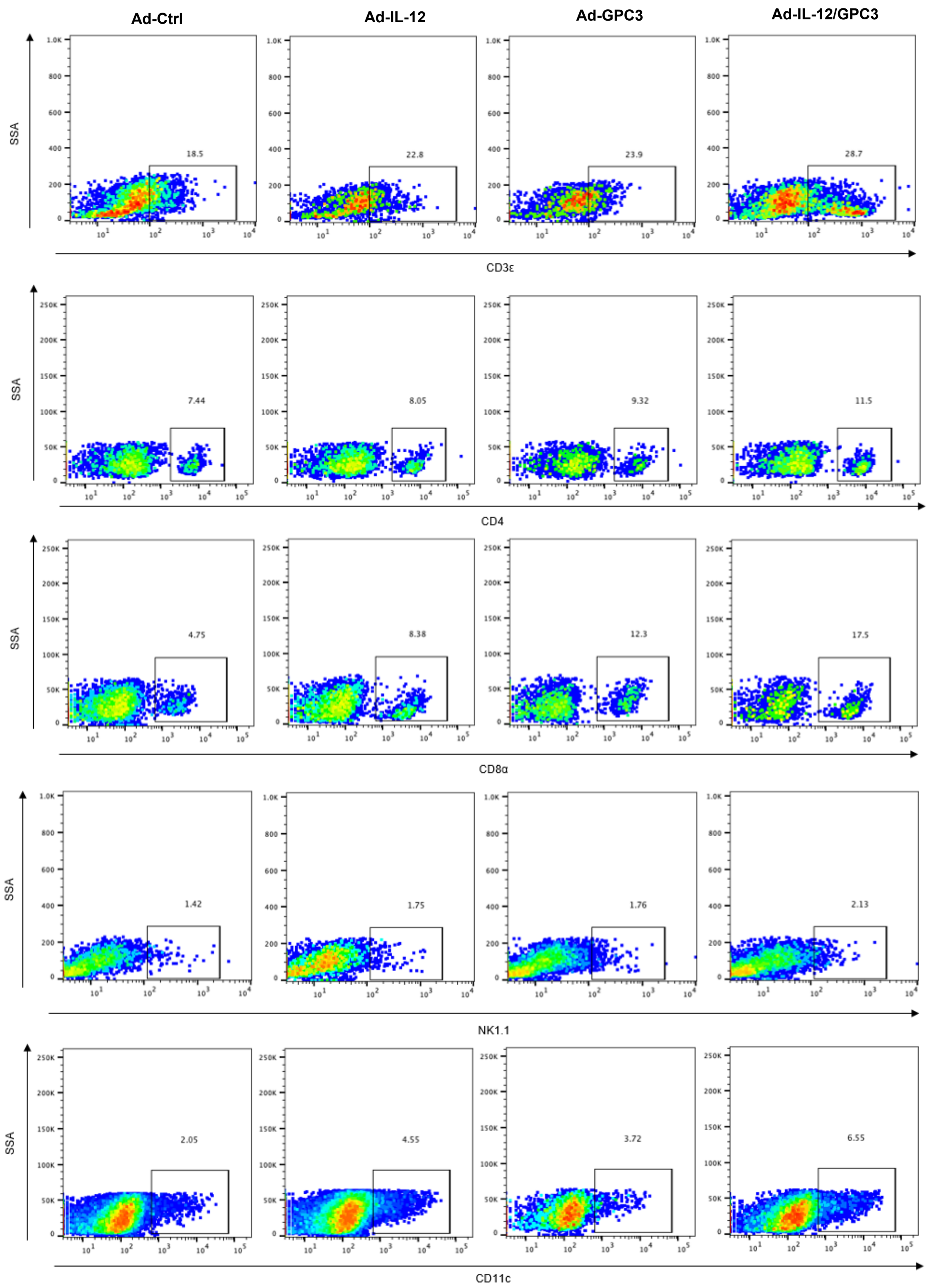

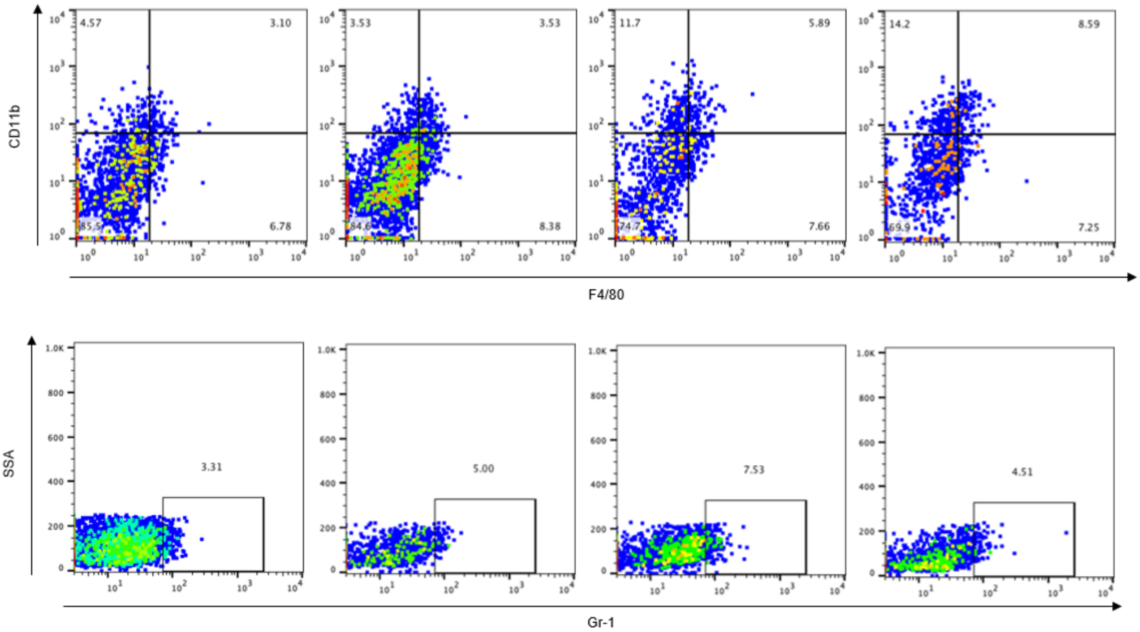

Mice were sacrificed on the 42nd day after tumor inoculation, and the frequencies of T, CD4^{+} T, CD8^{+} T, NK, DCs, macrophages, or MDSCs were analyzed in TILs of mice. Results from one representative experiment are shown for each group.
