# Supplementary material for: Therapeutic Adenovirus Vaccine Combined Immunization with IL-12 Induces Potent CD8+ T Cell Anti-Tumor Immunity in Hepatocellular Carcinoma
Source: Cancers (Basel). 2022 Sep 17;14(18):4512. doi: 10.3390/cancers14184512 (PMC9497125; doi:10.3390/cancers14184512)
Supplement: Supplementary file 1 [file cancers-14-04512-s001.zip › Figure S3.pdf]

Figure. S3. The percentages of $CD8^{+}CD11c^{+}$ cells were detected in DCs from lung tumor by flow cytometry.

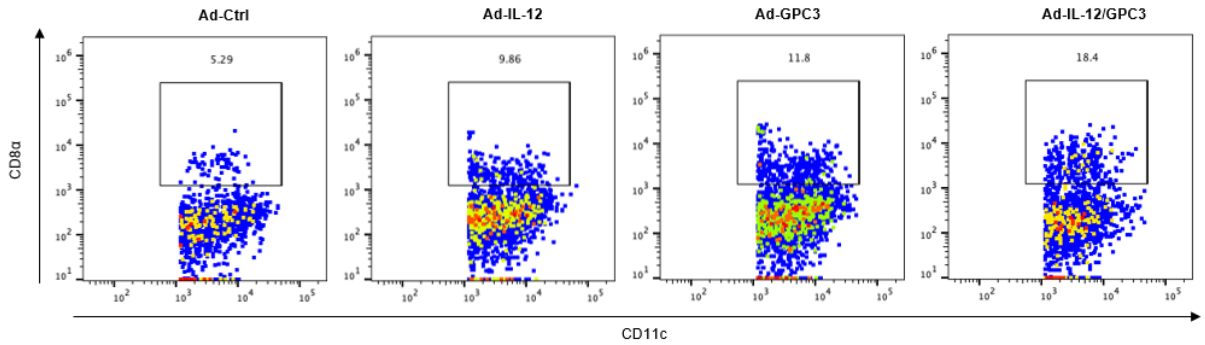

Mice were sacrificed on day 28 after tumor inoculation, and the frequencies of $CD8^{+}CD11c^{+}$ cells by gating $CD11c^{+}$ cells in TILs from the lung tumor tissues of various vaccine groups were analyzed. Each group showed the results of a representative experiment.
