# Supplementary material for: Therapeutic Adenovirus Vaccine Combined Immunization with IL-12 Induces Potent CD8+ T Cell Anti-Tumor Immunity in Hepatocellular Carcinoma
Source: Cancers (Basel). 2022 Sep 17;14(18):4512. doi: 10.3390/cancers14184512 (PMC9497125; doi:10.3390/cancers14184512)
Supplement: Supplementary file 1 [file cancers-14-04512-s001.zip › Table S1.pdf]

Table S1: The densitometry readings/relative intensity of each band of the densitometry scans.

	Lan1-1	Lan2-1	Lan1-2	Lan2-2	Lan1-3	Lan2-3
m-IL-12	23.942	41.955	25.143	59.012	24.897	55.786
Actin	119.958	116.661	132.765	124.500	117.865	151.642
m-IL-12/Actin	0.200	0.360	0.189	0.474	0.211	0.368
Lanx/Lan1	1.000	1.802	0.800	2.002	1.200	2.090

	Lan1-1	Lan2-1	Lan1-2	Lan2-2	Lan1-3	Lan2-3
GPC3	161.218	145.410	169.514	154.895	195.812	176.765
GAPDH	6.052	73.950	6.516	98.970	5.786	65.830
GPC3/GAPDH	0.038	0.509	0.038	0.639	0.030	0.372
Lanx/Lan1	0.890	12.057	1.000	16.622	1.110	13.990
